# Nitrate-Melt Synthesized HT-LiCoO_2_ as a Superior Cathode-Material for Lithium-Ion Batteries

**DOI:** 10.3390/ma2030857

**Published:** 2009-07-27

**Authors:** Mariyappan Sathiya, Annigere S. Prakash, Kannadka Ramesha, Ashok K. Shukla

**Affiliations:** 1 Central Electrochemical Research Institute, Karaikudi-630 006, Tamil Nadu, India; E-Mail: sathyamariyappan@gmail.com (M.S.); Prakash.as@gmail.com (A.P.); rkannadka@yahoo.com (K.R.); 2 Solid State and Structural Chemistry Unit, Indian Institute of Science, Bangalore-560 012, India

**Keywords:** LiCoO_2_, synthesis, nitrate decomposition, Li-ion batteries

## Abstract

An electrochemically-active high-temperature form of LiCoO_2_ (HT-LiCoO_2_) is prepared by thermally decomposing its constituent metal-nitrates at 700 ºC. The synthetic conditions have been optimized to achieve improved performance with the HT-LiCoO_2_ cathode in Li-ion batteries. For this purpose, the synthesized materials have been characterized by powder X-ray diffraction, scanning electron microscopy, and galvanostatic charge-discharge cycling. Cathodes comprising HT-LiCoO_2_ exhibit a specific capacity of 140 mAhg^-1^ with good capacity-retention over several charge-discharge cycles in the voltage range between 3.5 V and 4.2 V, and can sustain improved rate capability in contrast to a cathode constituting LiCoO_2_ prepared by conventional ceramic method. The nitrate-melt-decomposition method is also found effective for synthesizing Mg-/Al- doped HT-LiCoO_2_; these also are investigated as cathode materials for Li-ion batteries.

## 1. Introduction

Lithium cobalt oxide (LiCoO_2_) remains the most exploited cathode material for commercial Li-ion batteries [1]. LiCoO_2_ cathode exhibits high output-voltage, high specific-energy, long cycle-life and low self-discharge features that are central to batteries powering portable-electronic devices [1,2,3,4]. Electrochemical performance of LiCoO_2_ greatly depends on its crystallographic structure, as it exists in two different modifications, namely the high-temperature (HT) phase, crystallizing in an ideal layered-structure isomorphic to α-NaFeO_2_ (space group: R3m) with ordered cobalt and lithium ions resulting in hexagonal sheets of Li^+^- and Co^3+^- ions in alternate layers of (111) planes [5], and the low-temperature (LT) phase with spinel-like structure (space group: Fd3m) with about 6 % of Co^3+^ ions located at lithium sites [6]. Unlike LT-LiCoO_2_, HT-LiCoO_2_ exhibits excellent electrochemical stability on prolonged cycling [7,8]. The stability of HT phase originates from the structural durability of the material with the layered cation-ordering that remains well preserved even after repeated insertion and de-insertion of Li^+^-ions during the charge-discharge processes of the lithium-ion cell.

The structure and degree of cation ordering in LiCoO_2_ vary with the synthetic conditions that affect its electrochemical activity. Accordingly, the optimization of synthetic procedure for LiCoO_2_ is seminal for attaining its improved electrochemical behavior. To this end, various synthetic methods, such as ceramic method [9], oxalate method [10], hydroxide precipitation method [11], sol-gel method [12], molten salt method [13], hydrothermal method [14], template method [15], spray pyrolysis [16], polymer pyrolysis method [17], Pechini method [18] and combustion method [19], have been attempted in the literature for realizing electrochemically active LiCoO_2_. 

This communication reports a rapid synthesis of LiCoO_2_ cathode material with high specific-capacity for Li-ion batteries. The method involves heating stoichiometric amounts of metal-nitrate precursors that on decomposing yield a crystalline oxide. Since the metal nitrates have low melting-point, their mixtures transform to a eutectic melt on heating to the eutectic temperature that on further heating yields nano-crystalline LiCoO_2_ with perfect layered-structure suitable for use as active cathode material in a lithium-ion rechargeable battery. The method is also useful for synthesizing Mg-/Al-ions doped LiCoO_2_ and related intercalation oxides. It is noteworthy that the present method is altogether novel and differs substantially from other nitrate-decomposition methods [18,19,20]. The method is simple and cost-effective for bulk synthesis of HT-LiCoO_2_.

## 2. Experimental

Powder samples of LiCoO_2_ were prepared by rapidly heating the mixture of LiNO_3_ and Co(NO_3_)_2_.6H_2_O in the molar ratio of 1.1:1 with an excess of lithium stoichiometry (10 mol %) added to compensate for the lithium losses during the synthesis. In a typical preparation, 3.87 g of LiNO_3_ and 14.87 g of Co(NO_3_)_2_·6H_2_O were taken in a sintered alumina crucible and introduced to a preheated furnace at 350 ºC. Subsequently, the furnace was programmed for rapid heating to 700 ºC and held there for 1 h. The furnace was then fast cooled to 350 ºC in about 10 min and the crucible removed from the furnace. The sample was crushed and ground well to obtain a fine powder of LiCoO_2_. Similarly, Mg- or Al- doped HT-LiCoO_2_ with respective compositions of LiCo_0.95_Mg_0.05_O_2_ and LiCo_0.95_Al_0.05_O_2_ were prepared by taking corresponding metal nitrates in the required stoichiometry with 10 mol % extra lithium nitrate, followed by heating at 700 ºC for 1h. For comparison, HT-LiCoO_2_ was also prepared using the conventional solid-state method [9]; this sample is referred to as LiCoO_2_-SS in the text. 

Powder X-ray diffraction patterns for the prepared samples were recorded using X’pert PRO-PANalytical Diffractometer with CuK_α_ radiation. The morphology of the powder samples were examined under a Scanning Electron Microscope (HITACHI Model S-3000H). Chemical analyses of the samples were carried out using Perkin-Elmer Atomic Absorption Spectrometer (AAS). Electrochemical tests were conducted using Swagelok-type^TM^ cells assembled in an argon-filled glove box. The positive electrode comprised the ball-milled mixture of 85 wt.% active material with 15 wt.% Super-P Li Carbon (Timcal Belgium) as the conducting additive. Lithium electrode was prepared by pressing a piece of lithium metal onto a thin stainless steel disc. Electrolyte used was 1M LiPF_6_ solution in a mixture of ethylene carbonate and dimethyl carbonate in 1:1 ratio by volume. The cells thus fabricated were cycled galvanostatically between 3.5 V and 4.3 V versus lithium using a VMP3Z (Biologica) multi-channel potentiostat/galvanostat.

## 3. Results and Discussion

**Figure 1:**
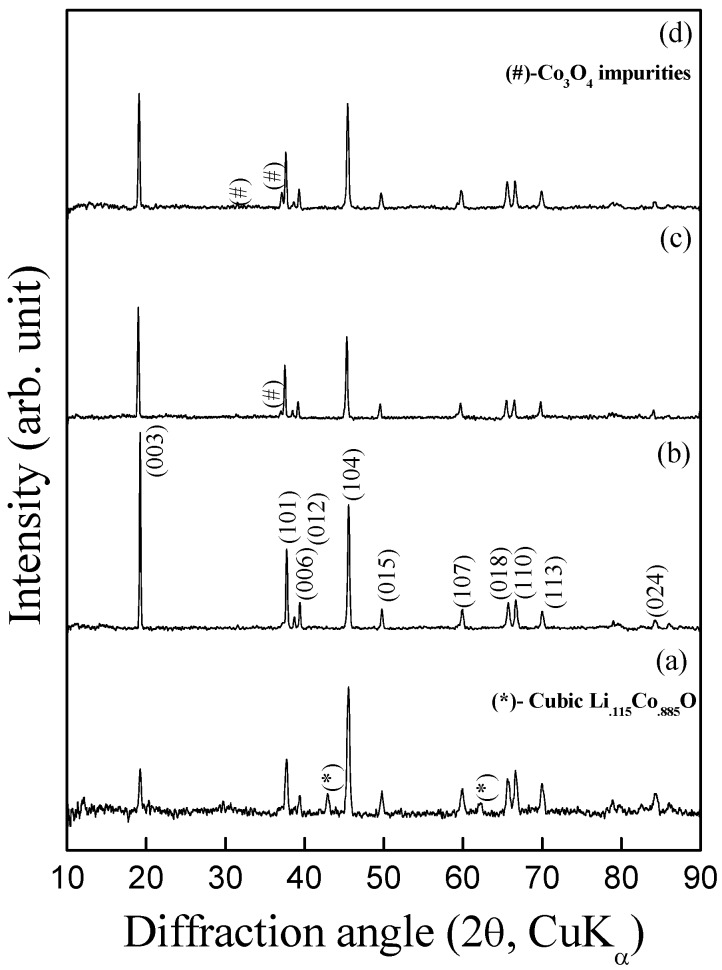
Powder X-ray diffraction patterns for LiCoO_2_ obtained by nitrate melt decomposition at varying temperatures (a) 600 ºC (b) 700 ºC (c) 800 ºC (d) 900 ºC. The peaks marked as (#) correspond to Co_3_O­_4_ and (*) correspond to lithium-deficient phase, Li_0.115_Co_0.885_O (ICSD collection code: 029229).

Figures 1(a-d) show the powder X-ray diffraction patterns for the products formed by heating the mixed-metal nitrates of lithium and cobalt in a 1.1:1 molar ratio at varying temperatures between 600 ºC and 900 ºC for 4 h. The diffraction pattern for the product obtained at 600 ºC could be indexed as rhombohedral R3m LiCoO_2_ along with small amounts of lithium-deficient LiCoO_2_ crystallizing in cubic Fm3m structure. X-ray powder diffraction pattern for the product obtained by heating at 700 ºC shows single-phase LiCoO_2_ as indexed on the basis of α-NaFeO_2_ structure (space group: R3m). 

The lattice parameters obtained by Rietveld refinement of LiCoO_2_-700 sample agree well with those prepared by conventional solid-state method [21] and are given in Table 1. All diffraction patterns show clear (006)/(102) peaks and (018)/(110) split peaks indicating a perfect layered-structure for LiCoO_2_ [21]. The diffraction patterns of samples prepared at 800 ºC and 900 ºC comprise a major LiCoO_2_ phase crystallizing in rhombohedral structure with a small Co_3_O_4_ spinel phase marked with (*) in Figure 1 (c) and (d); the latter arising due to lithium evaporation from the parent compound at high temperatures. I_(003)_/I_(104)_ intensity ratio decreases with increasing calcination temperature and is sensitive to the degree of cation mixing [22] that influences electrochemical properties substantially. 

**Table 1:** Lattice parameters, c/a ratio and I_(003)_/I_(104)_ intensity ratio for HT-LiCoO_2_ related phases.

Sample	*a* (Å)	*c* (Å)	*c*/*a*	I_(003)_/I_(104)_
LiCoO_2_-700	2.8132(8)	14.0630(3)	4.998	1.58
LiCoO_2_-SS	2.8164(6)	14.0568(7)	4.991	1.79
LiCo_0.95_Mg_0.05_O_2_	2.8172(6)	14.0770(5)	4.996	1.27
LiCo_0.95_Al_0.05_O_2_	2.8116(8)	14.0702(6)	5.004	1.14

**Figure 2:**
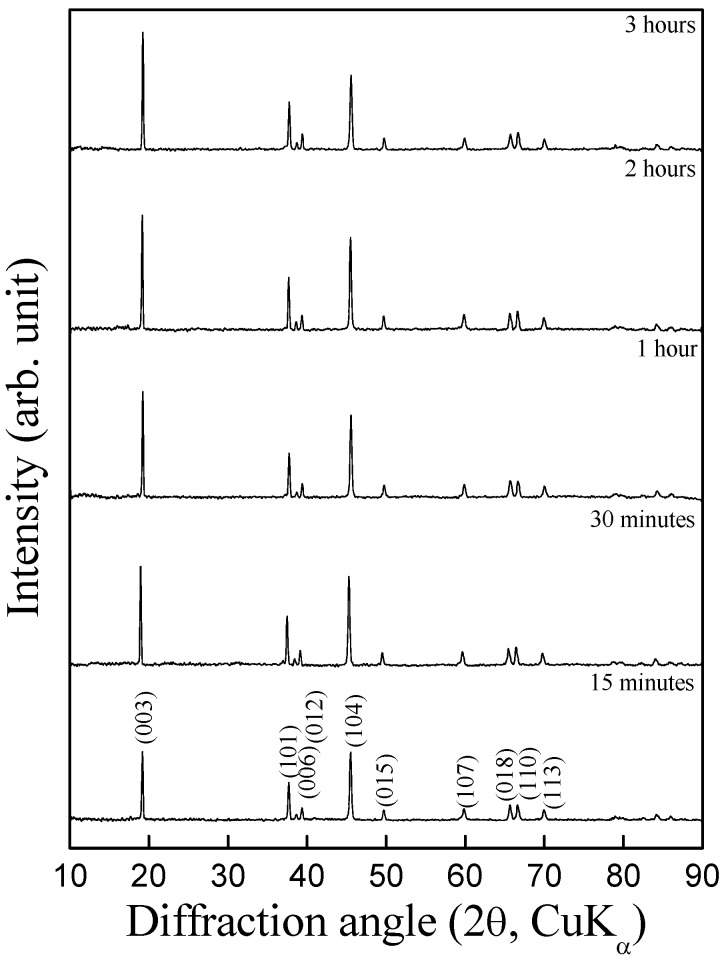
Powder X-ray diffraction patterns for LiCoO_2_ obtained by heating nitrates at 700 °C for varying durations.

It is clear from the aforesaid X-ray diffraction studies that 700 ºC is the optimal temperature for obtaining pure LiCoO_2_ in ordered rock-salt superstructure. In order to determine the optimal duration, the synthesis was carried out with varying heating durations between 15 min and 4 h at 700 ºC. The X-ray diffraction patterns for the products formed at different time intervals are presented in Figure 2. It is seen from the diffractograms that the sample prepared at 700 ºC for as low as 15 min matches well with HT-LiCoO_2_ pattern with R3m space group; the intensity ratio of I_(003)_/I_(104)_ <1.2 indicating a considerable extent of cation mixing in the crystal lattice. By contrast, the samples synthesized at 700 ºC for 1 h (or more) exhibit I_(003)_/I_(104)_ >1.2 indicating the absence of cation mixing. On increasing the heating duration beyond 1 h, little change is observed in the diffraction pattern with increase in overall peak intensity owing to the improved crystallinity of the sample. 

**Figure 3:**
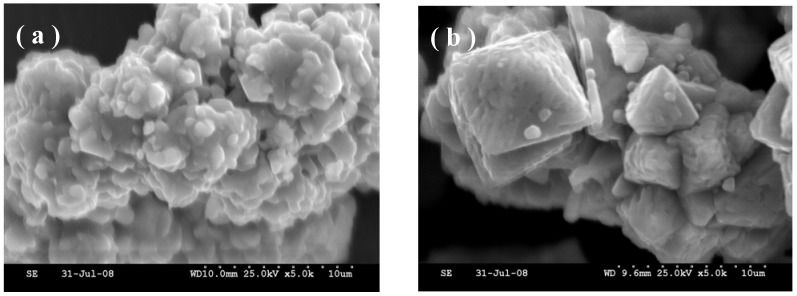
Scanning Electron Micrographs for HT-LiCoO_2_ obtained by heating corresponding metal nitrates at 700 °C in air for (a) 1 h and (b) 4 h.

The increase in crystallinity of the sample with longer heating duration is further corroborated from morphological studies by scanning electron microscopy (SEM). The SEM image for the samples heated at 700 ºC for 1 h shown in Figure 3(a) depicts agglomerates of smaller crystallites of ~1 μm. The sample obtained by heating at 700 ºC for 4 h shows well defined crystals of platelet-like morphology of ~ 50 μm [Figure 3(b)]. The chemical compositions of the prepared samples obtained from AAS analysis of Li and Co suggest cation stoichiometry to be strongly dependent on the synthesis temperature. LiCoO_2_ prepared at 600 ºC shows a slight excess of lithium stoichiometry corresponding to Li_1.09_CoO_2_ and closure to the initial stoichiometry of nitrates taken, suggesting little loss of Li during heating. It is noteworthy that impurity peaks corresponding to lithium salts are absent in the X-ray diffractogram. The sample prepared at 700 ºC has its nominal composition as LiCoO_2_ while the samples prepared at 800 ºC and above reflect Co_3_O_4_ as impurity in the diffractogram albeit the use of 10 mol % excess Li salt in the synthesis. This suggests substantial amount of Li loss at high temperatures. Chemical analyses of these samples were not possible owing to the insolubility of Co_3_O_4_ in acids. From the fore going, it is clear that the synthetic conditions have a seminal role in controlling structural as well as compositional aspects of LiCoO_2_ that affect its electrochemical activity. 

**Figure 4:**
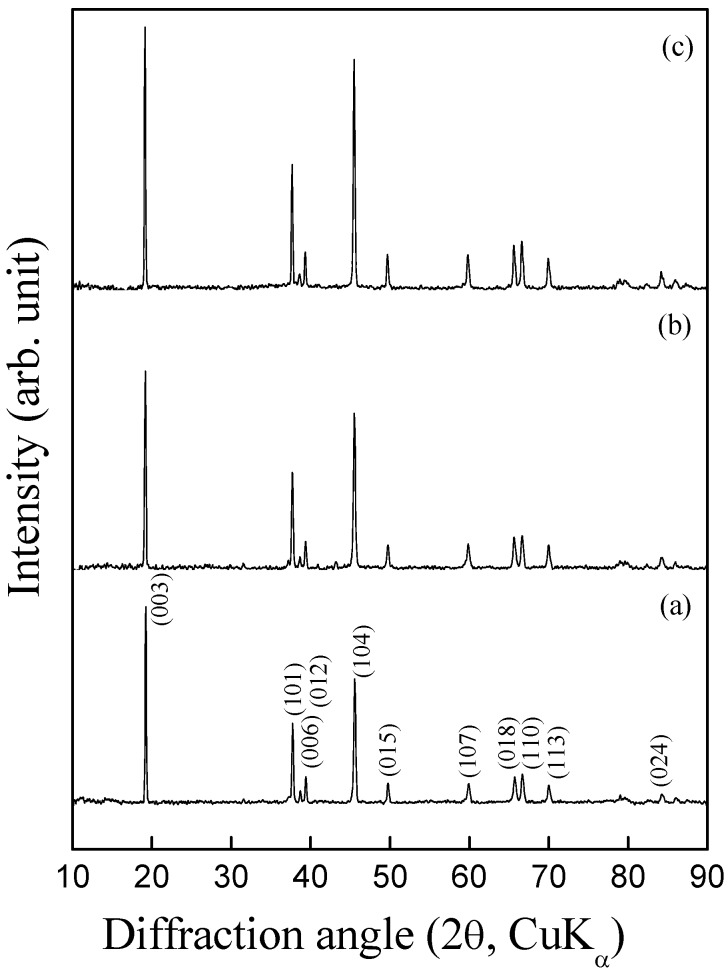
Powder X-ray diffraction patterns for (a) pristine LiCoO_2,_ (b) LiCo_0.95_Mg_0.05_O_2_ and (c) LiCo_0.95_Al_0.05_O_2_.

The metal-nitrates-melt decomposition method has added advantage in relation to solution methods as the former results in homogeneously mixed metal ions leading to their effective substitution in the end product. It has been possible to synthesize about 5-6 atom % of Mg- or Al- doped LiCoO_2_. Figure 4 shows powder X-ray diffraction patterns for LiCo_0.95_Mg_0.05_O_2_ and LiCo_0.95_Al_0.05_O_2_ samples along with the XRD pattern for pristine LiCoO_2_. All these XRD patterns could be indexed to a layered α-NaFeO_2_ structure (space group: R3m). The samples are phase pure and no impurity peaks corresponding to Li_2_CO_3_ and metal oxides are present. The refined lattice parameters for LiCo_0.95_Mg_0.05_O_2_ and LiCo_0.95_Al_0.05_O_2_ are presented in Table 1 which agrees well with those reported in the literature for similar compositions [23,24]. Substitution of Co^3+^ by larger Mg^2+^-ion increases the *a* and *c* lattice parameters with *c*/*a* ratio of 4.99 while substitution by smaller Al^3+^-ion decreases the *a*-lattice parameter and increases the *c*-lattice parameter resulting in an increased *c*/*a* ratio of 5.004.

**Figure 5:**
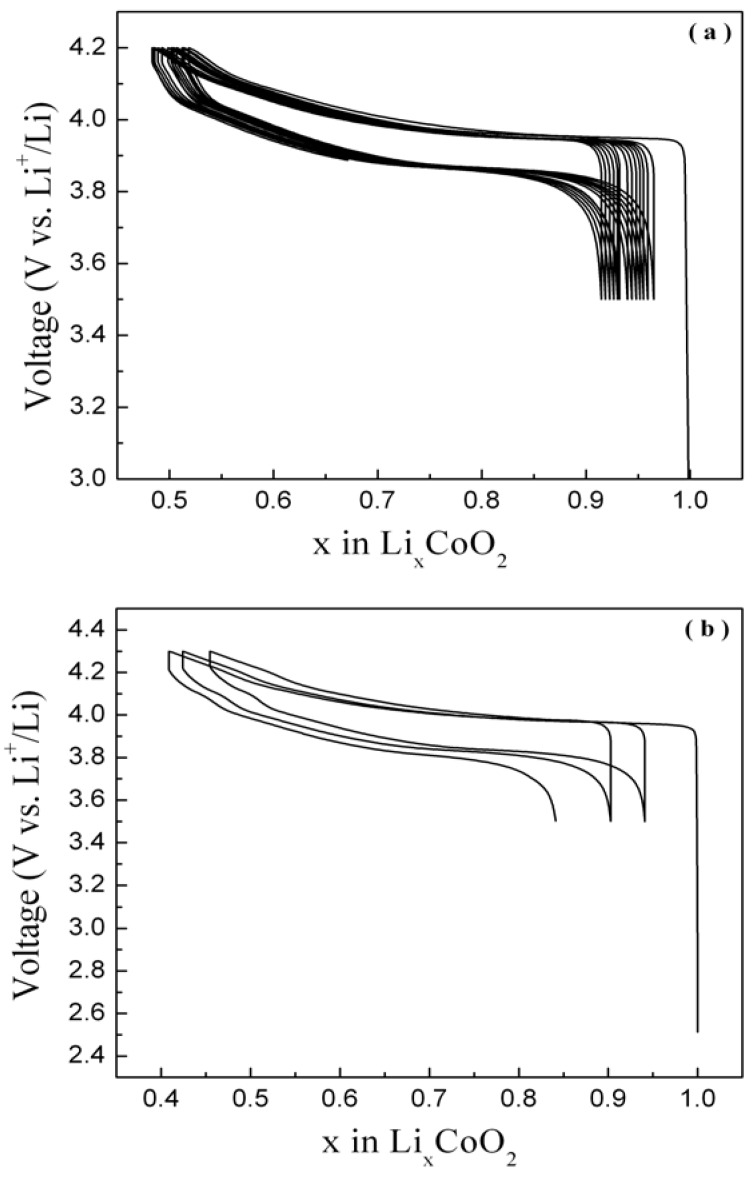
The voltage-composition curve for a Li/Li_x_CoO_2_-700 cell cycled galvanostatically at 1 Li/5 h rate between the voltage window (a) 3.5 - 4.2 V and (b) 3.5 - 4.3 V at room temperature (~30 ºC).

The electrochemical performances of all the LiCoO­_2_ samples, prepared at different temperatures with varying heat durations, have been evaluated using Swagelok-type cells. The voltage-composition curves for galvanostatic charge/discharge cycling of Li/LiCoO_2_-700 cells at 1 Li/5 h in different voltage ranges are shown in Figures 5(a) and (b) as representative examples. 

The smooth charge-discharge curves between 3.5 V and 4.2V show the absence of spinel-phase formation during cycling with the plateau due to Co^3+^/Co^4+^ redox process at ~3.9 V. A higher capacity of 150 mAhg^-1^ is obtained when the cells are cycled between 3.5 V and 4.3 V but the irreversible capacity loss, polarisability and capacity fade are higher compared to cells cycled between 3.5 V and 4.2 V. This is probably due to the change in crystal structure associated with the higher lithium extraction during higher voltage windows.

**Figure 6:**
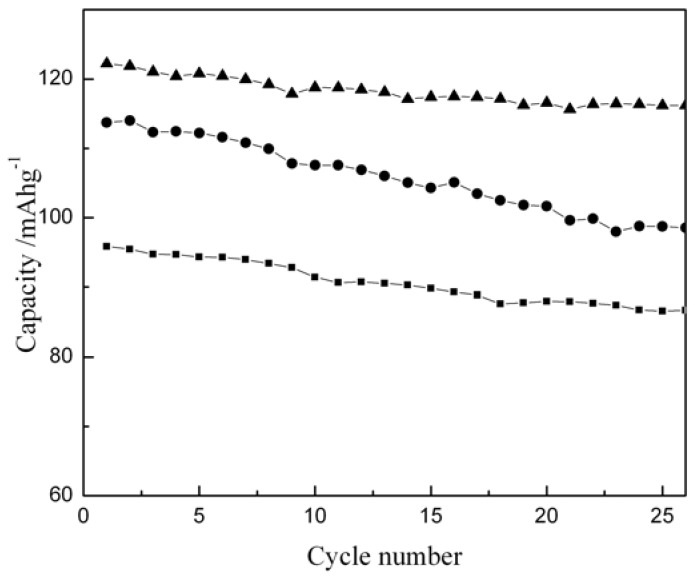
Discharge capacity versus cycle number for Li/LiCoO_2_ cells cycled between 3.5-4.2 V at room temperature ( ~30 ºC ) with LiCoO_2_ prepared at different temperatures: -●- 800 ºC, -▲- 700 ºC and -■-600 ºC.

Figure 6 shows the specific capacity values versus cycle number for Li/LiCoO_2_ cells with LiCoO_2_ samples prepared at different temperatures. The first charge/discharge capacities of the samples prepared at 600 °C, 700 °C and 800 °C are 106/91, 132/122 and 131/114 mAhg^-1^, respectively. As obvious from the data, the sample obtained by heating at 600 ºC shows lower capacity and retains only 92% of its initial capacity after 25 cycles, presumably due to the absence of perfect-layered structure as revealed by its X-ray diffraction pattern. By contrast, the sample prepared at 700 °C shows a maximum capacity with an irreversible capacity loss of about 10 mAh/g; the sample retains a capacity value of ~ 116mAhg^-1^ after 25 cycles with a capacity loss of only ~ 5%. The initial charge capacity for the sample synthesized at 800 °C is comparable to the sample prepared at 700 °C but its irreversible capacity loss in the first cycle is 17 mAhg^-1^ that pushes its specific capacity to ~114 mAhg^-1^. This is due to the Li-deficiency in the sample brought about by lithium loss at higher temperature. Accordingly, the sintering temperature clearly affects electrochemical behavior of the samples. In this study, the optimum electrochemical performance is observed for stoichiometric LiCoO_2_ prepared at 700 °C. 

**Figure 7:**
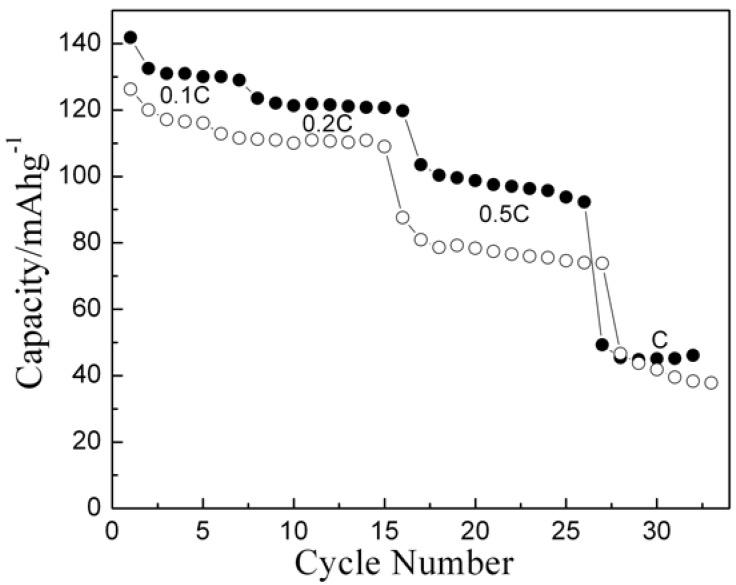
Rate capability plot for Li/LiCoO_2_ cells for nitrate derived (-●-) and solid-state prepared (-○-) samples.

In order to compare the electrochemical performance of HT-LiCoO_2_ prepared by nitrate-melt method with conventionally prepared samples, the cells of LiCoO_2_ prepared by solid state (LiCoO_2_-SS) and nitrate-melt (LiCoO_2_-700) methods have been subjected to cycling at varying rates. The capacity Vs cycle number plot is shown in Figure 7. The data reflect that the sample derived by nitrate-melt method exhibits higher capacity and better rate capability as compared to the sample prepared by conventional ceramic method. 

**Figure 8:**
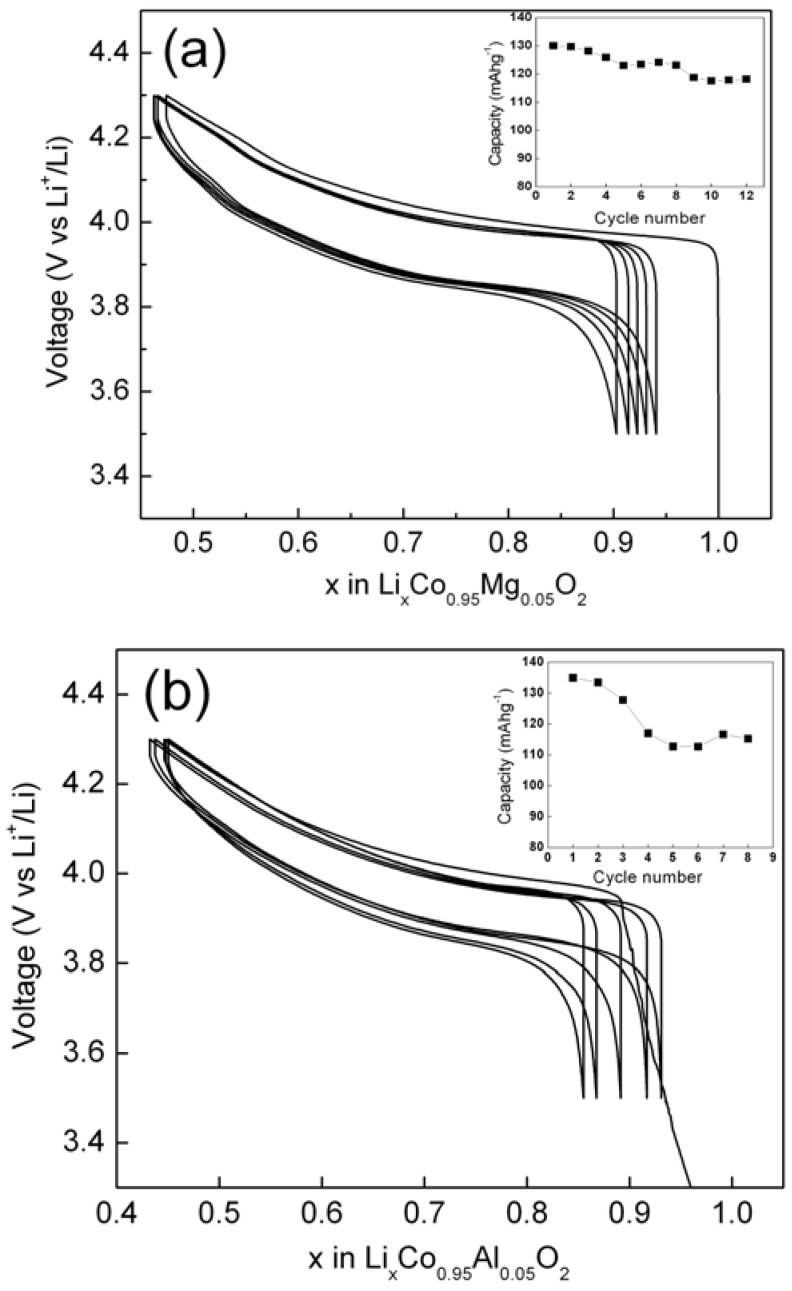
The voltage versus composition curves for (a) Li/LiCo_0.95_Mg_0.05_O_2_ and (b) Li/LiCo_0.95_Al_0.05_O_2_ cells in the voltage window 3.5-4.3 V. Corresponding capacity versus cycle number data are shown as insets.

Further, we have also evaluated electrochemical performances of Mg- or Al-doped LiCoO_2_ samples prepared by nitrate-melt-decomposition method. Figures 8(a) and (b) show the voltage versus composition curves for Li/LiCo_0.95_Mg_0.05_O_2_ and Li/LiCo_0.95_Al_0.05_O_2_ cells, respectively. The corresponding plots of capacity versus cycle number are shown as insets to Figures 8 (a) and (b).

The Mg-doped samples show good reversibility during charge/discharge cycling with first cycle capacity as high as 146 mAhg^-1^, a value comparable to un-doped LiCoO_2_. As expected, Mg doping enhances the structural stability with good capacity retention between 3.5 V and 4.3 V in relation to pristine LiCoO_2_. Interestingly, Li/LiCo_0.95_Al_0.05_O_2_ cell shows very high initial capacity of ~ 154 mAhg^-1^ but has poor capacity retention

We have demonstrated that nitrate-melt-decomposition route leads to electrochemically active pristine and doped LiCoO_2_. Although, synthesis of electrochemically active LiCoO_2_ through nitrate precursors followed by combustion, sol-gel or flame pyrolysis methods have been reported previously [18,19,20], these methods require a fuel or chelating agent in addition with metal precursors. Generally, the product obtained using these methods have residual carbonaceous matter which requires high-temperature heating for long duration. Besides, in many cases, the product formed is amorphous and requires further calcination for crystallization. In this context, the metal-nitrate-melt-decomposition route is attractive and new. The method is simple as well as cost effective for synthesizing battery grade LiCoO_2_ in bulk. 

## 4. Conclusions

Electrochemically active HT-LiCoO_2_ has been prepared by heating nitrate precursors for duration as short as 15 min. LiCoO_2_ prepared using this method exhibits varying degrees of cation mixing and crystallinity depending on the synthetic conditions. The samples prepared at 700 ºC for shorter durations are nano-crystalline in nature and exhibit increased rate capability. On increasing the reaction temperature above 700 ºC, samples are found to be lithium deficient. HT-LiCoO_2_ obtained by nitrate-melt decomposition at 700 ºC results in a superior cathode material for lithium-ion batteries. The method is also effective for synthesizing Mg- or Al- doped LiCoO_2_. The nitrate-melt decomposition method is simple, cost effective and convenient for large-scale synthesis of LiCoO_2_ with better electrochemical performance.
